# Pathogenic and Antigenic Analyses of H5N1 High Pathogenicity Avian Influenza Virus Isolated in the 2022/2023 Season From Poultry Farms in Izumi City, Japan

**DOI:** 10.1155/tbed/1535116

**Published:** 2025-02-23

**Authors:** Hayate Nishiura, Asuka Kumagai, Miki H. Maeda, Yoshihiro Takadate, Saki Sakuma, Ryota Tsunekuni, Junki Mine, Yuko Uchida, Kohtaro Miyazawa

**Affiliations:** ^1^Emerging Virus Group, Division of Zoonosis Research, National Institute of Animal Health, National Agriculture and Food Research Organization, Tsukuba, Ibaraki 305-0856, Japan; ^2^Plant Resources Unit, Research Center of Genetic Resources, National Agriculture and Food Research Organization, Tsukuba, Ibaraki 305-8602, Japan

## Abstract

During the winter of 2022/2023, Japan experienced its largest outbreak of high pathogenicity avian influenza (HPAI), affecting 84 poultry premises. In this study, we investigated the pathogenicity and antigenicity of A/chicken/Kagoshima/22A1T/2022 (Kagoshima/22A1T), a clade 2.3.4.4b H5N1 virus belonging to the G2b group. It was isolated from a poultry farm in Izumi City, where the largest number of consecutive cases was recorded. The 50% lethal dose, mean death time (MDT), amount of virus shed, and transmissibility in chickens of Kagoshima/22A1T were similar to those of A/chicken/Kagoshima/21A6T/2022 (Kagoshima/21A6T), the previous season's isolate of the same group, indicating that their pathogenicities were comparable. However, the antigenicity of these isolates differed according to the hemagglutination inhibition (HI) test results. We found that the amino acid substitutions in residues 189 and 193, corresponding to antigenic site B in the H3 virus of the HA1 subunit, could have an impact on the HI cross-reactivity of Kagoshima/21A6T. This study provides important insights into the factors contributing to the consecutive HPAI outbreaks on poultry farms in Izumi City during the 2022/2023 season and the prediction of antigenic changes in G2b group HPAI viruses.

## 1. Introduction

High pathogenicity avian influenza viruses (HPAIVs) of the H5Nx subtype circulate in poultry and wild birds worldwide. The hemagglutinin (HA) genes of these viruses, derived from the A/goose/Guangdong/1/1996 (Gs/Gd/96) [1] lineage, have diversified into multiple clades (0–9), with some clades further divided into subclades [2]. The clade 2.3.4.4, which is predominantly in circulation worldwide, has been subdivided into subclades a–h [3]. In 2020, clade 2.3.4.4b viruses spread predominantly through migratory birds to many African, Asian, and European countries, resulting in an extraordinary number of bird and poultry deaths [4]. In 2021, the virus spread to North America and in 2022 to Central and South America [4]. Currently, interspecies transmission has also been highlighted as a wide range of terrestrial and marine mammals which have been reported with clade 2.3.4.4b virus infection in several countries [5–7]. Notably, in the United States, more than 800 confirmed cases of the H5N1 virus in dairy cattle have been reported in 16 states as of December 19, 2024 (US Department of Agriculture, online). This may mark a new phase in which the H5N1 virus of clade 2.3.4.4b threatens the global panzootic. Consequently, understanding outbreaks in avian populations—the natural reservoirs of the virus—has become more critical than ever.

**Figure 1:**
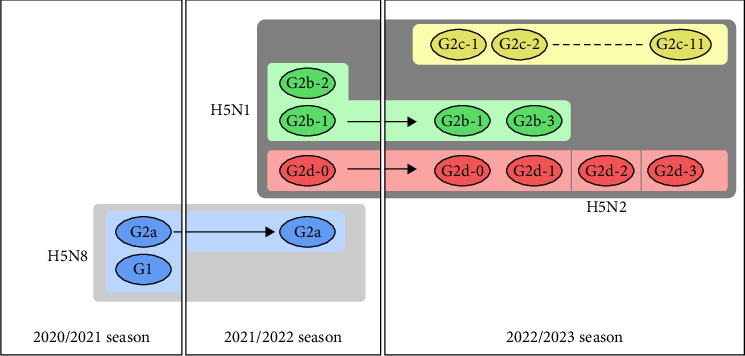
The recent trend of high pathogenicity avian influenza virus (HPAIV) groups in Japan. In the 2020/2021 season, there were only two groups, G1 and G2a, derived from the European isolates. In the 2021/2022 season, two new groups, G2d and G2b, were identified in addition to the G2a group. In the following season, in addition to the outbreaks of the G2d-0 and G2b-1 genotypes, three genotypes of G2d and G2b-3 and 11 genotypes of a new group, G2c, were confirmed.

In Japan, high pathogenicity avian influenza (HPAI) outbreaks due to clade 2.3.4.4b H5Nx viruses have recently been increasing at an unprecedented pace in poultry farms and wild birds. In the winter of 2020/2021, there were 52 outbreaks at poultry farms and 58 outbreaks in wild birds or environmental water samples [8–10]. Subsequently, in the 2021/2022 season (i.e., from November 2021 to May 2022), 25 outbreaks were reported in poultry premises, and 107 cases were confirmed in wild birds [11]. Eventually, in the 2022/2023 season (i.e., from September 2022 to April 2023), there were 84 outbreaks in poultry premises and 242 cases in wild birds or environmental water samples, the largest number ever recorded in Japan. Phylogenetic analyses revealed that the 2.3.4.4b H5Nx HPAIV isolates in Japan from the 2020/2021 season to the 2022/2023 season comprised five distinct genetic groups in total (G1, G2a, G2b, G2c, and G2d) [12–14] (Figure 1). In previous studies, our research team referred to G2a, G2b, G2c, and G2d as 20A, 20E, 21RC, and 21E, respectively; however, we now apply the former nomenclature mentioned above based on the consensus of Japanese researchers in all institutes conducting definitive HPAI diagnosis. Based on the phylogenetic analysis of eight gene segments including the HA gene, the 2.3.4.4b H5Nx HPAIVs isolated from poultry farms in the 2022/2023 season were classified into 17 genotypes [15] (Figure 1).

**Figure 2:**
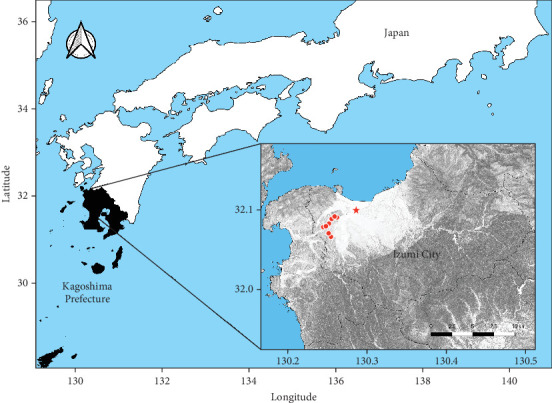
Location of the farms where the high pathogenicity avian influenza (HPAI) outbreak occurred during the 2022/2023 season. The white area in the northwest of Izumi City in the close-up map corresponds to the Izumi Plain. The nine sites (a red star and red circles) from which G2b-1 group viruses were isolated are located within an 8 km radius. A red star represents the farm where Kagoshima/22A1T was isolated. The remaining eight sites are in hilly areas.

Poultry farms in Kagoshima Prefecture had the highest number of HPAI outbreaks in the 2022/2023 season, with 13 consecutive cases (11 in the G2b-1 group and two in the G2c-8 group) in 2 months. Nine of the 11 outbreaks of the G2b-1 virus occurred in Izumi City and two occurred in Akune City, both of which are very close to the Izumi Plain (an area in the northwest part of Izumi City) (Figure 2). The Izumi Plain is a Ramsar site, which is a wetland of international importance providing habitats for many migratory birds, including endangered cranes (hooded cranes and white-naped cranes) and waterfowl carrying HPAIV [16, 17]. Izumi has a thriving poultry industry and is one of the leading egg producers in Japan. Therefore, this region has attracted the attention of many avian influenza researchers, and there is an urgent need to elucidate the factors that caused consecutive HPAI outbreaks in poultry farms during the 2022/2023 season. Here, we analyzed the pathogenicity and antigenicity of HPAIV isolated from a farm on the Izumi Plain in the 2022/2023 season and compared the results with those of a previous study on a 2021/2022 isolate.

## 2. Materials and Methods

### 2.1. Virus Isolation

The Kagoshima Central Livestock Hygiene Service Center obtained tracheal and cloacal swab fluids from layer chickens (547 days old) that died at a poultry farm in Kagoshima Prefecture and were diagnosed with HPAIV infection using H5-specific qPCR. The swab fluids were inoculated into 9–11-day-old embryonated chicken eggs, and allantoic fluids were collected. The fluids were submitted to our laboratory for further analysis. Genome sequencing was performed as previously described [8, 18]. The isolated virus was designated as A/chicken/Kagoshima/22A1T/2022 (H5N1) and referred to as Kagoshima/22A1T in this study. The amino acid sequence of the HA cleavage site of Kagoshima/22A1T was estimated to be PLRERRRKR/GLFG, a characteristic HPAIV motif [19], suggesting that Kagoshima/22A1T is a high pathogenicity isolate. Nucleotide sequences are available in the GISAID database (http://platform.gisaid.org; EPI_ISL_18286302).

### 2.2. Animal Experiments

Animal experiments were approved by the Institutional Committee for Ethics of Animal Experiments (approved number R4-I014-NIAH-5) and conducted in biosafety level 3 facilities at the National Institute of Animal Health. The animal experiments were designed based on previous reports [8–11] and carried out in compliance with the ARRIVE guidelines. We used 32 four-week-old White Leghorn chickens obtained from Nisseiken Co., Ltd. (Tokyo, Japan). Serum was collected from all birds before virus inoculation, and ELISA was performed using a virus antibody test kit (IDEXX Influenza A Ab Test; IDEXX Laboratories, Westbrook, ME) to confirm that all chickens were seronegative for influenza A virus. To evaluate the 50% chicken lethal dose (CLD_50_), isolator-reared chickens were randomly divided into five groups (five chickens each) and were each inoculated intranasally with 100 μL of Kagoshima/22A1T with doses ranging from 10^2^ to 10^6^ 50% egg infectious dose (EID_50_) (referred to as the lethality study henceforth). The infected chicken mean death time (MDT) was calculated from the results of the 10^6^ EID_50_-inoculated group. In a virus transmission study, to ensure infection and to compare the results of a previous study [11], a chicken was inoculated with 10^6^ EID_50_ of Kagoshima/22A1T. One day later, six chickens were housed with the inoculated chicken. We clinically monitored all chickens and collected tracheal and cloacal swabs at 1, 2, 3, 5, 7, 10, and 14 days postinoculation (dpi) or when the chickens died. Sampling was performed in the order of low to high EID_50_-inoculation groups with thorough ethanol disinfection before and after each sampling. The infected chickens were euthanized by intravascular injection of an overdose of pentobarbital at the end of the experiment or when the humane endpoint was reached. The collected swabs were suspended in a medium prepared as previously described [11]. The swab fluids were inoculated into embryonated eggs to measure the infectious viral titer, and the EID_50_ was calculated using the Reed–Muench method [20]. The detection limit was 0.2 log_10_ EID_50_/mL. An ELISA was performed to determine whether the chickens surviving at 14 dpi were infected.

### 2.3. Hemagglutination Inhibition (HI) Tests

The antigenic relationships of HPAIV isolates were evaluated using the cross-HI test with standard protocols provided by the World Health Organization for animal influenza diagnosis and surveillance using antiserum against HPAIVs [21]. The HI titer value is the inverse of the last dilution of serum that completely inhibited hemagglutination. The antisera against A/chicken/Kagoshima/21A6T/2022 (Kagoshima/21A6T [Kagoshima6T in a previous report [11]]; GISAID database, EPI_ISL_6829533), which was isolated in the 2021/2022 season and belongs to the G2b-1 group, as well as the antisera against A/chicken/Iwate/21A7T/2022 (Iwate/21A7T [Iwate7T in the previous report]), used in this study were previously produced by our research team [11]. We produced antisera against Kagoshima/22A1T and A/chicken/Kagawa/22A9T/2022 (Kagawa/22A9T) by immunizing chickens four times with 1.0 mg of the respective inactivated antigens with Freund's incomplete adjuvant (Sigma-Aldrich, St. Louis, MO) (approval number 23-067). The viruses used as antigens in this study were as follows: A/chicken/Kagoshima/21A1T/2021 (Kagoshima/21A1T), A/chicken/Kagoshima/21A2T/2021 (Kagoshima/21A2T), A/chicken/Kagoshima/21A3T/2021 (Kagoshima/21A3T), A/chicken/Kagoshima/21A4T/2021 (Kagoshima/21A4T), Kagoshima/21A6T, A/chicken/Hiroshima/21A10C/2021 (Hiroshima/21A10C), Kagoshima/22A1T, A/emu/Fukuoka/22C2T/2023 (Fukuoka/22C2T), A/chicken/Oita/22A4T/2023 (Oita/22A4T), and A/chicken/Kagoshima/22M3T/2023 (Kagoshima/22M3T).

### 2.4. Construction of Model Structures of HA

Molecular modeling including homology modeling was performed using MOE 2020.0901 with default parameters. The best rank model of 10 candidates for each strain was employed for further analysis. The template 3D structure was the A subunit of 7DEB (7DEB-A) in the Protein Data Bank.

### 2.5. Statistical Analysis

The difference in viral titers between the tracheal and cloacal samples collected in this experiment was evaluated by the Wilcoxon signed-rank test using the statistical software EZR (Saitama Medical Center, Jichi Medical University, Saitama, Japan) [22]. Statistical significance was set at *p* < 0.05.

## 3. Results

### 3.1. Pathogenicity of Kagoshima/22A1T in Chickens (the Lethality Study)

**Figure 3:**
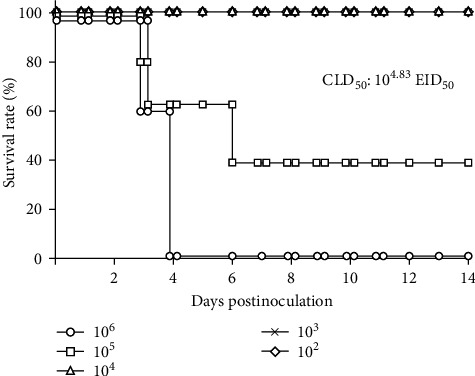
Survival rates of chickens inoculated Kagoshima/22A1T. The survival rates of chickens inoculated with 10^2^, 10^3^, 10^4^, 10^5^, and 10^6^ EID_50_ of each virus are shown by rhombuses, crosses, triangles, squares, and circles, respectively.

**Figure 4:**
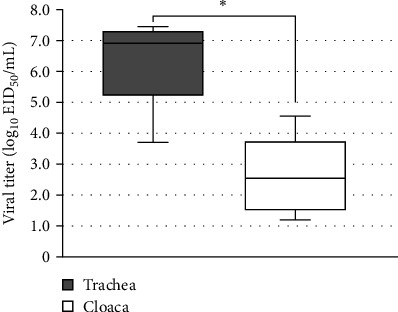
Boxplot of viral titer in tracheal and cloacal swabs of the carcasses of chickens inoculated with the 10^6^ EID_50_ of Kagoshima/22A1T. Chickens (*n* = 5) were nasally inoculated with 10^6^ EID_50_ of Kagoshima/22A1T. *⁣*^*∗*^*p* < 0.05; Wilcoxon signed-rank test.

To estimate the MDT and CLD_50_ values, we intranasally inoculated Kagoshima/22A1T into chickens and observed them daily for 14 days in the lethality study. At 2 dpi, all chickens inoculated with 10^6^ EID_50_ showed lethargy, depression, and anorexia (Supporting Information 1: Figure S1); two died at 3 dpi, and the other three died at 4 dpi. The MDT calculated for the 10^6^ EID_50_ inoculated group was 3.7 days (88.8 h) (Table 1). Four chickens showed cyanosis (subcutaneous hemorrhage and necrosis) in their combs and legs and edema in their combs and eyelids (Table 1 and Supporting Information 2: Figure S2). One of the five chickens inoculated with 10^5^ EID_50_ showed signs of depression, and three died within 5 dpi (Supporting Information 1: Figure S1); however, no gross lesions were observed. Sera from the two surviving chickens did not show significant anti-influenza A antibodies in ELISA at 14 dpi. Chickens inoculated with 10^2^, 10^3^, and 10^4^ EID_50_ survived for 14 days (Figure 3) without obvious clinical signs and tested negative by ELISA. The CLD_50_ of Kagoshima/22A1T was calculated to be 4.83 log_10_EID_50_ per chicken (Table 1). The mean viral titers of tracheal and cloacal swab samples collected from the carcasses of chickens inoculated with 10^6^ EID_50_ are shown in Figure 4. The mean viral titer of tracheal swabs was significantly higher (6.35 ± 0.67 log_10_EID_50_) than that of cloacal swabs (2.60 ± 0.56 log_10_EID_50_) (Table 1) based on the Wilcoxon signed-rank test (*p* < 0.05).

### 3.2. Transmissibility of Kagoshima/22A1T in Chickens (the Transmission Study)

In the transmission study, the virus-inoculated chicken died at 3 dpi (Supporting Information 3: Figure S3) with subcutaneous hemorrhage of the leg and hemorrhagic necrosis of the comb, similar to that described in the lethality study. Three of the six cohabiting chickens died within 7 dpi, meaning 6 days postcohabitation (dpc), but no significant clinical signs or gross lesions were observed. The remaining three survived to the end of the experiment and tested negative by ELISA for antibodies against the influenza A virus, indicating that they were not infected. The transmissibility was calculated as 50% (Table 1).

**Figure 5:**
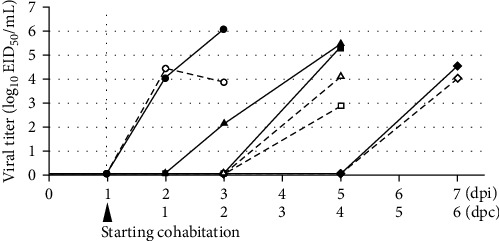
Kinetics of viral titers in each tracheal and cloacal swab. Black and white circles represent the viral titer in the tracheal and cloacal swabs, respectively, from the chicken inoculated with the 10^6^ EID_50_ of Kagoshima/22A1T. Black triangles, squares, and rhombuses represent the viral titer in the tracheal swabs of the cohabiting chickens, while white triangles, squares, and rhombuses represent the viral titer in the cloacal swabs of the cohabiting chickens.

Viral titer kinetics in the tracheal and cloacal swabs are illustrated in Figure 5. Virus-inoculated chicken shed the virus into the trachea and cloaca at 2 dpi. The viral titers in the trachea and cloaca increased until death to a level similar to that observed in the lethality study (Figure 4). At 3 dpi (2 dpc), one cohabiting chicken shed the virus from the trachea, whereas at 5 dpi (4 dpc), two cohabiting chickens died and shed the virus from the trachea and cloaca. One of the remaining chickens died at 7 dpi (6 dpc) and shed 4.54 and 4.02 log_10_EID_50_/mL virus from the trachea and cloaca, respectively.

### 3.3. Antigenic Analyses of H5 Subtype Isolates

To examine antibody reactivity, an HI test was conducted with antigens prepared from seven isolates from different years and/or belonging to different groups (Table 2). The antiserum against Kagoshima/22A1T exhibited relatively low HI titers against Kagoshima/21A6T, which were eightfold lower than those against the homologous antigen. Antiserum against Kagoshima/21A6T, isolated in the 2021/22 season and belonging to the G2b-1 group, only showed high reactivity with its homologous antigen, not with the others. Antisera against Iwate/21A7T and Kagawa/22A9T cross-reacted with most of the antigens tested, but their HI titers against Kagoshima/21A6T were four- and twofold lower than those of the others, respectively.

A comparison of the HA amino acids of the virus used for the HI test revealed an amino acid substitution, E189K, according to H3 numbering [27], in the receptor-binding site of Kagoshima/21A6T, which was not found in the other isolates (Supporting Information 5: Table S1). In addition, amino acid substitutions 193A and 193D were identified in Kagoshima/22A1T and Kagoshima/21A6T, respectively. An additional study was conducted to determine whether these amino acid substitutions significantly affected their antigenicity. We investigated five additional viruses (Supporting Information 6: Table S2): Kagoshima/21A1T, Kagoshima/21A2T, Kagoshima/21A3T, and Kagoshima/21A4T, which were isolated from the same farm at the same time as Kagoshima/21A6T in 2021 and had no E189K substitutions in their HA1, and Hiroshima/21A10C, which had an E189K substitution. Antisera against Kagoshima/21A6T strongly cross-reacted with Kagoshima/21A6T and Hiroshima/21A10C (Table S2). In contrast, HI titers against the other isolates were remarkably low, especially against Kagoshima/22A1T, which has two amino acid differences at the antigenic site. Antiserum with an HI titer to Kagoshima/22A1T of 1280 showed titers against Kagoshima/21A1T and Hiroshima/21A10C of 160.

### 3.4. Comparison of Four Modeled Structures of HA

**Figure 6:**
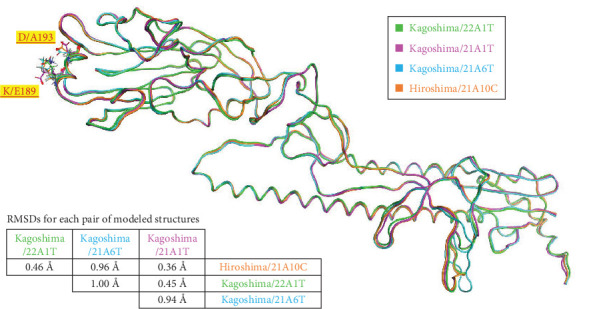
Main chains of modeled influenza hemagglutinin (HA) molecules (Kagoshima/22A1T, green; Kagoshima/21A1T, magenta; Kagoshima/21A6T, cyan; and Hiroshima/21A10C, orange). Root mean square distance (RMSD) of α-carbons among the four models was 0.749 Å.

The 7DEB-A was chosen as a template for homology modeling because the sequence identity between Kagoshima/22A1T and 7DEB-A is 98% and coverage is 89%. Four model structures of Kagoshima/22A1T, Kagoshima/21A1T, Kagoshima/21A6T, and Hiroshima/21A10C were generated. When the structures were superimposed, the root mean square distances (RMSDs) of each pair of the models were between 0.36 and 1.00Å (Figure 6) about their backbones. This implied that the overall structures are almost the same.

The spatial positions of the residues 189 and 193 are shown in Figure 6. These residues are located on the outermost layer of the viral envelope, close to each other, and oriented outward on an α-helix. When zooming in on the detailed structures from the 185^th^ to 200^th^ residues (Supporting Information 4: Figure S4), there are no interactions between E189 and A193 in the Kagoshima/22A1T model (Supporting Information 4: Figure S4a). The opposite orientation of the side chains of E189 and D193 in the model of Kagoshima/21A1T is due to the electrostatic repulsion between the two side chains (Supporting Information 4: Figure S4b). Of note, the models of Kagoshima/21A6T and Hiroshima/21A10C indicate that the side chain atoms of K189 and D193 could form a hydrogen bond (Supporting Information 4: Figure S4c,d). This would stabilize the shape of an α-helix.

**Figure 7:**
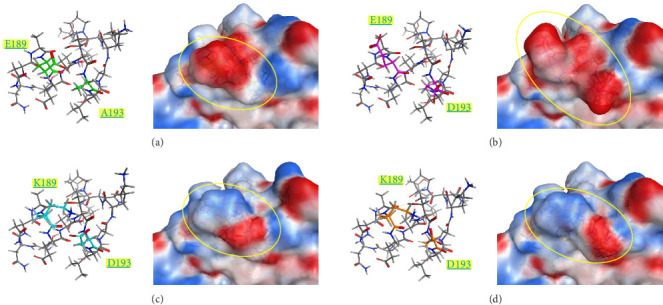
Surface environment of hemagglutinin (HA) of four isolates: (a) Kagoshima/22A1T, (b) Kagoshima/21A1T, (c) Kagoshima/21A6T, and (d) Hiroshiima/21A10C. The left panel shows the structure from P/S185 to T200, and the right panel shows the molecular surface in each figure. Colored residues are K/E189 and D/A193. K/E189 and D/A193 are shown as stick models of the same color in Figure 6, and the other residues from P185 to T200 are drawn as line models. Red, white, and blue areas on each electrostatic surface mean positive, neutral, and negative charge, respectively.

The surface charge distribution of each model is shown in Figure 7. Kagoshima/22A1T features a negative charge on the 189^th^ residue but no charge (neutral) on the 193^rd^ residue (Figure 7a). In the Kagoshima/21A1T model, the negative charge area expands on the 189^th^ and 193^rd^ residue surface area (Figure 7b). In contrast, Kagoshima/21A6T and Hiroshima/21A10C have a positively charged area on the 189^th^ residue due to Lys in addition to a negative charge on the 193^rd^ residue (Figure 7c,d). Thus, these models indicate that the charge distribution of the HA molecular surface is significantly altered by the combination of the 189^th^ and 193^rd^ amino acid residues.

## 4. Discussion

During the 2022/2023 season, Japan experienced its largest HPAI outbreak, with Kagoshima Prefecture having the highest number of outbreaks [28]. Therefore, we focused on the outbreaks in Izumi City, a major overwintering area for migratory birds in Japan, and characterized one of the isolates from an outbreak farm as Kagoshima/22A1T.

The current study demonstrated that chickens inoculated with Kagoshima/22A1T showed cyanosis but did not display any neurological signs. Cyanosis has been commonly observed in infection studies on clade 2.3.4.4b isolates from Japan [8, 9, 11]. Although no detailed autopsy was performed, the direct cause of death in this study appears to be circulatory and respiratory failure, as hemorrhage and edema suggest an abnormality in blood circulation.

In the case of HPAI, because all infected chickens die, CLD_50_ corresponds to 50% of the chicken infectious dose; therefore, CLD_50_ represents the infectivity of HPAIVs. This means that MDT can be used as an indicator of HPAIV virulence in chickens. It had been reported that chickens inoculated with H5 subtype viruses belonging to clade 2.3.4.4b isolated from different regions show slightly variable MDT and CLD_50_ than expected (Table 1). This may be due to differences in NA and other internal gene combinations (the so-called genotype), as well as the genetic background of the chickens used in the experiments. Given that the isolates used in this study belong to the same genotype, it is natural for them to exhibit similar values for MDT and CLD_50_.

The viral shedding from the trachea of chickens inoculated with Kagoshima/22A1T was greater than that from the cloaca, which is consistent with the experimental results obtained with Kagoshima/21A6T [11] (Table 1). Comparing the results of the transmission studies of Kagoshima/22A1T and Kagoshima/21A6T [11], the infection rate was quite similar (both transmissibility were 50%) (Table 1). In the current study, virus shedding from the trachea occurred at most 2 days before death with titers of 2.1 log_10_EID_50_, less than one-hundredth of the CLD_50_. Viral titers from tracheal swabs at the time of death ranged from 4.5 to 6.0 log_10_EID_50_, indicating that the chickens may have been shedding virus comparable to the CLD_50_ for a maximum of ~24 h before death. The short period of viral shedding before death may have contributed to a transmissibility of 50%. It has been reported that some viruses belonging to clade 2.3.4.4b showed adaptation to ducks but were not efficiently transmitted to chickens [23, 29]. However, the transmissibility of HPAIVs belonging to the clade 2.3.4.4b varied from 0% to 100% as shown in Table 1. Furthermore, CLD_50_ also varied (maximum 250-fold difference). Therefore, it seems difficult to find a good correlation between transmissibility and CLD_50_ or transmissibility and MDT. Taken together, the possibility of chicken-to-chicken or farm-to-farm transmission cannot be ruled out based on the virological characteristics alone. Meanwhile, no epidemiological information is available at this time to support the transmission from farm to farm. Further studies will be required to determine the causes of consecutive HPAI outbreaks on poultry farms in Izumi City during the 2022/2023 season.

We conducted an HI cross-reactivity test to determine how the immunological aspects of Kagoshima/22A1T differed from those of the previous season and other group viruses in the 2022/2023 season. The antisera against Kagoshima/22A1T reacted with both the 2021/2022 and 2022/2023 season G2d isolates, as shown in Table 2. These results are consistent with those of a previous report that examined the cross-reactivity of 2021/2022 season isolates in Japan [13]. However, in a report using different G2a, G2b, and G2d isolates, antisera against the G2b isolate (Kagoshima/21A6T) showed little cross-reactivity with the G2d isolate (Iwate/21A7T), which was eightfold lower than the homologous titer [11]. To determine the cause of this difference, we compared the amino acid sequences of HA between the Kagoshima/22A1T and Kagoshima/21A6T isolates. We found that these two isolates had different amino acids at positions 189 and 193 of the HA1 subunit (Kagoshima/22A1T: 189E and 193A; Kagoshima/21A6T: 189K and 193D). Kaverin et al. [30] showed that one of the H5 antibody-escape mutants of H5N2 adapted to mice had a T189K substitution in the HA1 subunit but reported that this substitution did not affect immune specificity. The HI titer was the same between Kagoshima/22A1T and Kagoshima/21A6T for antisera against Kagawa/22A9T, which belonged to the G2c group and had an 189E. However, when we focused on the cross-reactivity of HPAIVs in the G2b group HI tests, the E189K substitution strongly affected immune specificity (Supporting Information 6: Table S2).

The 189^th^ residue is in a helix structure (188–195) in our model, which corresponds to the antigenic site B in the H3 virus [31]. In human seasonal influenza viruses (H3N2), the 189^th^ residue is one of the sites where an amino acid substitution can result in major antigenic changes [32]. Since residue 193 is in the spot corresponding to antigenic site B in the H3 virus, several studies have reported that amino acid changes at this position contribute to the antigenic variation of H5N1 viruses [30, 33, 34]. Indeed, our modeling analysis showed that the 189^th^ residue and the sterically neighboring 193^rd^ residue are at the outermost part of the viral envelope, making them easily accessible to antibodies. Therefore, they would significantly affect antibody reactivity.

Our modeled structures suggest differences in charge distribution on the surface of the HA molecule between the combination of the 189^th^ and 193^rd^ residues (Figure 7). The HA molecule of Kagoshima/21A1T has a combination of E189 and D193, corresponding to the expanded negative charge area. In contrast, Kagoshima/21A6T has a combination of K189 and D193, corresponding to positive and negative charge areas at the same site. Antigen recognition relies on the complementarity between antigen–antibody interfaces, and the electrostatic complementarity at an antigen–antibody interface is one of the critical factors determining the physical properties of antigen–antibody interaction [35, 36]. Therefore, it is assumed that the difference in surface charge distributions between Kagoshima/21A1T and Kagoshima/21A6T affected the difference in antigen–antibody interaction represented by the HI test. Furthermore, these modeled structures suggest that the difference between K (positive charge) and E (negative charge) at 189^th^ amino acid residues has a greater impact on the antibody–antigen response than the difference between D (negative charge) and A (uncharged) at 193^rd^ amino acid residues.

Although further investigation is required to determine why antisera against the G2c group HPAIV isolate possessing 189E shows cross-reactivity with the G2b group HPAIV isolate possessing 189K, our results will be of great help in predicting the antigenic differences of current epidemic viruses from the previous season's epidemic viruses based on amino acid sequences in group G2b HPAIVs.

Influenza viruses evolve easily through antigenic drift (mutation) and shift (reassortment of viral genome segments) [37]. The current study suggests that HPAIVs belonging to the G2b group have undergone antigenic drift, possibly owing to immune pressure and other factors. The Izumi Plain is the world's largest wintering area for hooded cranes and white-naped cranes [38, 39], but many other migratory birds besides cranes also visit this area. Consequently, the concentration of the virus in the environment increased during this time, which probably contributed to the consecutive outbreaks on the Izumi Plain during the 2022/2023 season. Because there are many poultry facilities in and around the Izumi Plain, the risk of HPAI outbreaks is considered very high, and it is important to continue to strengthen surveillance for wild birds.

## 5. Conclusion

We demonstrated that the infectivity, virulence, and transmissibility of H5N1 viruses classified as G2b-1 isolated from poultry farms in Izumi City were similar; however, their antigenicity has changed over the past 2 years. Our results provide significant insights into the causes of the second consecutive season of HPAIV outbreaks on poultry farms in Izumi City, and the prediction of antigenic changes in G2b group viruses.

## Figures and Tables

**Table 1:** Comparison of results of the lethality and transmission study in chickens inoculated with 2.3.4.4b H5 viruses isolated in 2020–2022.

Virus	Subtype	CLD_50_ (log_10_ EID_50_)	10^6^ EID_50_ inoculated chickens			Clinical signs	Transmissibility (%)	Reference
			MDT (days)	Mean viral titer^a^ (log_10_ EID_50_/mL)				
				Trachea	Cloaca			
Kagoshima/22A1T	H5N1	4.83	3.7	6.35	2.60	Lethargy, depression, anorexia, cyanotic comb and legs, edema in combs and eyelids	50	This manuscript
Kagoshima/21A6T	H5N1	4.50	3.3	5.97	3.47	Depression, neurological signs	50	[11]

A/chicken/Akita/7C/2021	H5N8	3.83	3.5	6.05	2.99	Depression, cyanotic comb and legs	100	[11]
A/chicken/Iwate/21A7T/2022	H5N1	4.68	2.2	8.75	7.51	Depression, neurological signs	33	[11]
A/chicken/Kagawa/11C/2020	H5N8	4.63	5.6	5.32	4.32	(Not described)	17	[9]
A/duck/Chiba/C1T/2021	H5N8	3.50	3.2	6.70	4.16	(Not described)	100	[9]
A/chicken/Fukuoka/T1/2020	H5N8	2.75	3.2	6.53	5.20	(Not described)	83	[9]
A/eastern buzzard/Toyama/160213T/2021	H5N8	4.50	4.4	6.32	5.20	(Not described)	100	[9]
A/chicken/Tokushima/4T/2020	H5N8	4.63	5.4	6.53	4.53	(Not described)	0	[9]

A/chicken/England/053052/2021	H5N1	4.67	3.1^b^	4.8^b,c,d^	4.4^b,c,d^	Huddling/ruffled feathers, lethargy, diarrhea, and neurological signs	0	[23]
A/chicken/England/030786/2020	H5N8	4.30	3.4	ND	ND	Huddling, ruffled feathers, dropped wings, and lethargy, neurological signs	20	[24]
A/mute swan/England/SA14-234255/2020	H5N1	5.00	2.2	ND	ND	Dropped wings, swelling, huddling, ruffled feathers, lethargy, and neurological signs	20	[24]
A/American wigeon/South Carolina/USDA-000345-001/2021	H5N1	2.60	1–2.7	6.5^c^	4.9^c^	Lethargy and/or unresponsive, ruffled feathers, periorbital swelling, and cyanotic combs	0	[25]
A/mandarin duck/Korea/H242/2020	H5N8	4.50	4.3	6.3	4.8	(Not described)	33	[26]

Abbreviation: ND, not done.

^a^The values of Kagoshima/22A1T represent the mean viral titer at the time of death, and the figures for Kagoshima/21A6T represent the mean maximum viral titer.

^b^The maximum dose in this study was 10^5^ EID_50_.

^c^Viral titers were determined by the M gene rRT-PCR method.

^d^The Ct values were converted to relative equivalent units (REUs/mL) for correlation with the EID_50_. As specific figures are not available in the reference, we estimated the REU values from the graphs in the reference.

**Table 2:** Serological cross-reactivity of three H5 virus groups (G2d, G2b, and G2c) isolated in Japan in the 2021/2022 and 2022/2023 seasons.

Antisera			Virus						
			2021/2022 season	2022/2023 season		2021/2022 season	2022/2023 season	2022/2023 season	
			G2d-0		G2d-2	G2b-1		G2c-1	G2c-8
			Iwate/ 21A7T	Fukuoka/22C2T	Oita/22A4T	Kagoshima/21A6T	Kagoshima/22A1T	Kagawa/22A9T	Kagoshima/22M3T
2021/2022	G2d-0	Iwate/21A7T	2560	2560	2560	640	1280	2560	2560
	G2b-1	Kagoshima/21A6T	<20	80	<20	2560	<20	<20	<20
2022/2023		Kagoshima/22A1T	1280	1280	320	160	1280	640	640
	G2c-1	Kagawa/22A9T	640	640	320	320	320	640	640

*Note:* Underlines represent the cross-reactivity to homologous antigens.

## Data Availability

The sequences generated in this study were submitted to the GISAID database with the accession number EPI_ISL_18286302.

## Supporting Information

Supporting Information 1 Figure S1: Detailed history of five chickens inoculated with 10^5^ and 10^6^ EID_50_ of Kagoshima/22A1T. Gray boxes, clinical signs (lethargy, depression, and anorexia); black boxes, the time of death; plus signs, gross findings (cyanosis) were observed; minus signs, no gross findings.

Supporting Information 2 Figure S2: Gross findings of the chicken inoculated with the 10^6^ EID_50_ of Kagoshima/22A1T.

Supporting Information 3 Figure S3: Detailed history of a chicken inoculated with 10^6^ EID_50_ of Kagoshima/22A1T and cohabiting chickens. Black boxes indicate the time of death. A plus sign indicates that there were gross findings at the time of death, and a minus sign indicates that there were no findings.

Supporting Information 4 Figure S4: Detailed structures from the 185th to the 200th residues of four models. (a) Superpose of four models, (b) Kagoshima/22A1T, (c) Kagoshima/21A1T, (d) Kagoshima/21A6T, and (e) Hiroshima/21A10C. Each color of the tube model corresponds to the drawing shown in Figure 7. Broken lines indicate hydrogen bonds.

Supporting Information 5 Table S1: Comparison of estimated amino acids in hemagglutinin of isolates used in the HI study.

Supporting Information 6 Table S2: Comparison of the estimated amino acid sequence of the HA1 gene and HI cross-reactivity of G2b-1 group viruses.
