# Supplementary material for: Pathogenic and Antigenic Analyses of H5N1 High Pathogenicity Avian Influenza Virus Isolated in the 2022/2023 Season From Poultry Farms in Izumi City, Japan
Source: Transbound Emerg Dis. 2025 Feb 23;2025:1535116. doi: 10.1155/tbed/1535116 (PMC12017051; doi:10.1155/tbed/1535116)
Supplement: Supporting Information 4 — Figure S4: Detailed structures from the 185th to the 200th residues of four models. (a) Superpose of four models, (b) Kagoshima/22A1T, (c) Kagoshima/21A1T, (d) Kagoshima/21A6T, and (e) Hiroshima/21A10C. Each color of the tube model corresponds to the drawing shown in Figure 7. Broken lines indicate hydrogen bonds. [file 1535116.f4.pptx]

## Slide 1
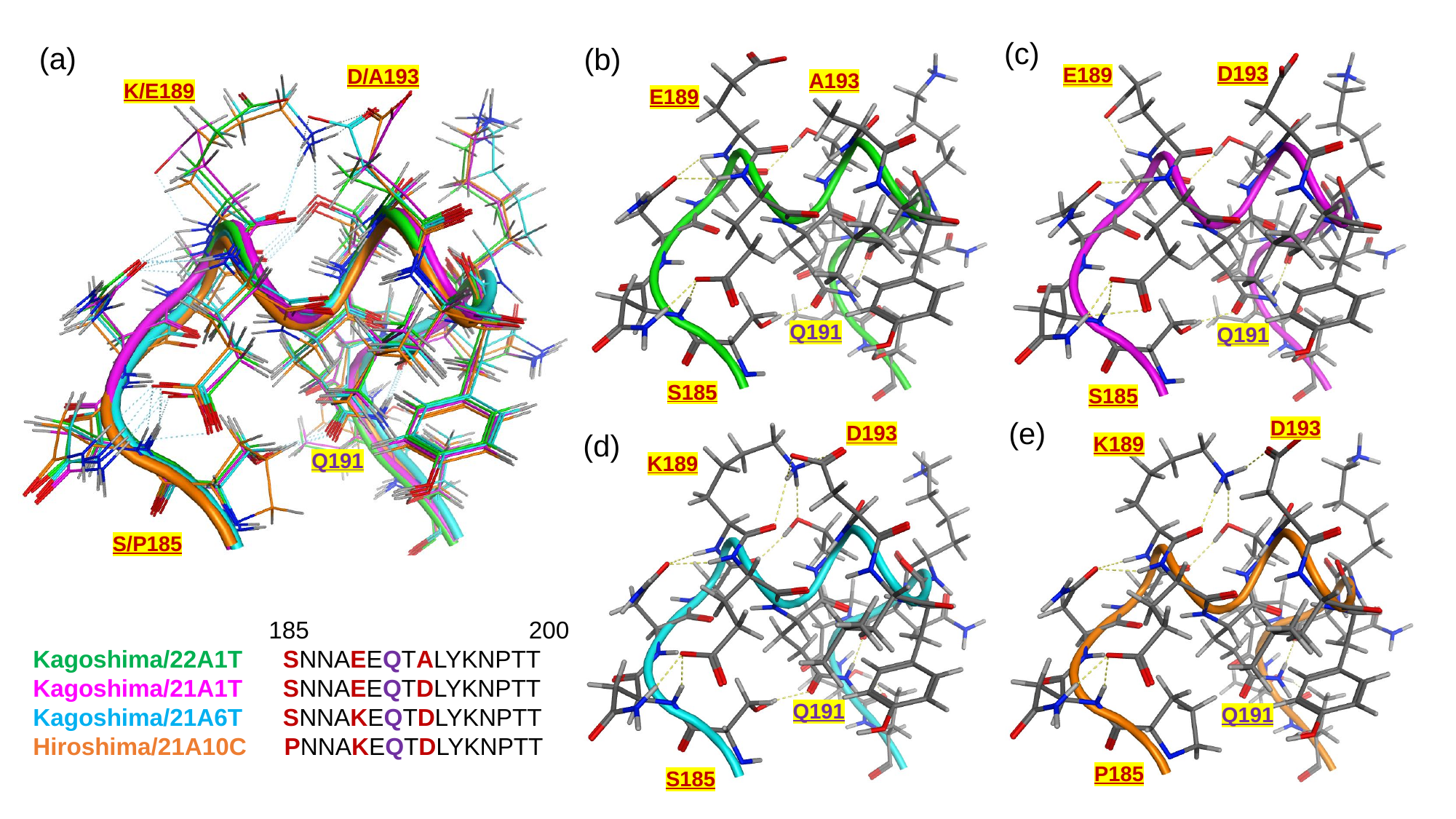

(c)
D193
E189
Q191
S185
(a)
(b)
A193
E189
Q191
S185
D/A193
K/E189
(e)
D193
K189
Q191
P185
D193
(d)
K189
Q191
S185
Q191
S/P185
 　　　　 185 　　　　　 200
Kagoshima/22A1T SNNAEEQTALYKNPTT
Kagoshima/21A1T SNNAEEQTDLYKNPTT
Kagoshima/21A6T SNNAKEQTDLYKNPTT
Hiroshima/21A10C PNNAKEQTDLYKNPTT
