# Supplementary material for: Pathogenic and Antigenic Analyses of H5N1 High Pathogenicity Avian Influenza Virus Isolated in the 2022/2023 Season From Poultry Farms in Izumi City, Japan
Source: Transbound Emerg Dis. 2025 Feb 23;2025:1535116. doi: 10.1155/tbed/1535116 (PMC12017051; doi:10.1155/tbed/1535116)
Supplement: Supporting Information 5 — Table S1: Comparison of estimated amino acids in hemagglutinin of isolates used in the HI study. [file 1535116.f5.docx]

| Table S1. Comparison of estimated amino acids in hemagglutinin of isolates used in the HI study. | | | | | | | | | | | | | | | | | | |
| --- | --- | --- | --- | --- | --- | --- | --- | --- | --- | --- | --- | --- | --- | --- | --- | --- | --- | --- |
|  |  |  | HA1 | | | | | | | | | | | | |  | HA2 | |
|  |  | H3 no. | 4 | 5 | 46 | 90 | 91 | － | － | 158 | 189 | 193 | － | 323 | 328 |  | 146 | 202 |
| Group | Viruses | H5 no. | － | － | 36 | 82 | 83 | 86 | 120 | 154 | 185 | 189 | 268 | 320 | 325 |  | 146 | 203 |
| G2d-0 | A/chicken/Iwate/21A7T/2022 | | I | I | T | R | A | A | S | N | E | N | G | S | R |  | N | M |
|  | A/emu/Fukuoka/22C2T/2023 | | － | － | － | － | － | － | － | － | － | － | － | － | － |  | D | － |
| G2d-2 | A/chicken/Oita/22A4T/2023 | | － | － | － | － | － | － | － | － | － | － | － | － | － |  | D | － |
| G2b-1 | A/chicken/Kagoshima/21A6T/2021 | | － | V | T | K | － | T | N | － | K | D | － | － | － |  | － | － |
|  | A/chicken/Kagoshima/22A1T/2022 | | － | V | T | K | － | T | N | － | － | A | E | － | － |  | － | L |
| G2c-1 | A/chicken/Kagawa/22A9T/2022 | | T | V | T | － | D | － | － | D | － | － | － | － | K |  | － | － |
| G2c-8 | A/chicken/Kagoshima/22M3T/2023 | | T | V | T | － | D | － | － | D | － | － | － | － | K |  | － | － |
