# Supplementary material for: Pathogenic and Antigenic Analyses of H5N1 High Pathogenicity Avian Influenza Virus Isolated in the 2022/2023 Season From Poultry Farms in Izumi City, Japan
Source: Transbound Emerg Dis. 2025 Feb 23;2025:1535116. doi: 10.1155/tbed/1535116 (PMC12017051; doi:10.1155/tbed/1535116)
Supplement: Supporting Information 6 — Table S2: Comparison of the estimated amino acid sequence of the HA1 gene and HI cross-reactivity of G2b-1 group viruses. [file 1535116.f6.docx]

Table S2: Comparison of the estimated amino acid sequence of the HA1 gene and HI cross-reactivity of G2b-1 group viruses

|  |  | 165 | | |  |  | 170 | | |  |  | 175 | | |  |  | 180 | | |  |  | 185 | | |  | 189 | | |  | 193 | | | 195 | |  |  | 200 | |  |
| --- | --- | --- | --- | --- | --- | --- | --- | --- | --- | --- | --- | --- | --- | --- | --- | --- | --- | --- | --- | --- | --- | --- | --- | --- | --- | --- | --- | --- | --- | --- | --- | --- | --- | --- | --- | --- | --- | --- | --- |
| **Kagoshima/22A1T** |  | I | K | I | S | Y | N | N | T | N | R | E | D | L | L | I | L | W | G | I | H | H | S | N | N | A | E | E | Q | T | **A** | L | Y | K | N | P | T | T |  |
| Kagoshima/21A1T |  | . | . | . | . | . | . | . | . | . | . | . | . | . | . | . | . | . | . | . | . | . | . | . | . | . | . | . | . | . | D | . | . | . | . | . | . | . |  |
| Kagoshima/21A2T |  | . | . | . | . | . | . | . | . | . | . | . | . | . | . | . | . | . | . | . | . | . | . | . | . | . | . | . | . | . | D | . | . | . | . | . | . | . |  |
| Kagoshima/21A3T |  | . | . | . | . | . | . | . | . | . | . | . | . | . | . | . | . | . | . | . | . | . | . | . | . | . | . | . | . | . | D | . | . | . | . | . | . | . |  |
| Kagoshima/21A4T |  | . | . | . | . | . | . | . | . | . | . | . | . | . | . | . | . | . | . | . | . | . | . | . | . | . | . | . | . | . | D | . | . | . | . | . | . | . |  |
| Kagoshima/21A6T |  | . | . | . | . | . | . | . | . | . | . | . | . | . | . | . | . | . | . | . | . | . | . | . | . | . | **K** | . | . | . | D | . | . | . | . | . | . | . |  |
| Hiroshima/21A10C |  | . | . | . | . | . | . | . | . | . | . | . | . | . | . | . | . | . | . | . | . | . | P | . | . | . | **K** | . | . | . | D | . | . | . | . | . | . | . |  |
|  |  |  |  |  |  |  |  |  |  |  |  |  |  |  |  |  |  |  |  |  |  |  |  |  |  |  |  |  |  |  |  |  |  |  |  |  |  |  |  |
| Antisera | G2b-1 group viruses | | | | | | | | | | | | | | | | | | | | | | | | | | | | | | | | | | | | | | |
|  |  | 2022/23 season | | | | |  | 2021/22 season | | | | | | | | | | | | | | | | | | | | | | | | | | | | | | |  |
|  |  |  |  |  |  |  |  | Isolates from the same farm at the same time | | | | | | | | | | | | | | | | | | | | | | | | |  | with E189K substitution | | | | |  |
|  |  | **Kagoshima /22A1T** | | | | |  | Kagoshima /21A1T | | | | | Kagoshima /21A2T | | | | | Kagoshima /21A3T | | | | | Kagoshima /21A4T | | | | | Kagoshima /21A6T | | | | |  | Hiroshima /21A10C | | | | |  |
| **Kagoshima/22A1T** |  | 1280 | | | | |  | 640 | | | | | 640 | | | | | 640 | | | | | 640 | | | | | 160 | | | | |  | 160 | | | | |  |
| Kagoshima/21A6T |  | 40 | | | | |  | 160 | | | | | 160 | | | | | 160 | | | | | 320 | | | | | 2560 | | | | |  | 2560 | | | | |  |

Underlines represent cross-reactivity to homologous antigens. The gray highlights represent amino acid changes at residues 189 and 193 and their contribution to reduced HI reactivity of the isolates.
